# Surgery

**Published:** 1826-04

**Authors:** 


### SURGERY.

19. *Carotid Aneurism.** There is no department of surgery in which the improvements introduced by British surgeons, have been more important or conspicuous, than in that which relates to the securing of arteries by ligatures, and more especially in the treatment of aneurism. Among the numerous benefits which mankind owe to the superior genius of John Hunter, there is none that redounds more to his honor than the simple, easy, and safe operation which he devised for the cure

* Case of Carotid Aneurism, successfully treated by tying the Artery above the Aneurismal Tumour. By James Wardrop, Surgeon Extraordinary to the King. From the Thirteenth Volume of the Medico-Chirurgical Transactions.

of popliteal aneurism, a disease which, previous to his time, had almost invariably ended fatally, or for which the operations known to surgeons had most frequently had only the effect of varying the mode, or accelerating the period of the patient's death. Mr. Hunter likewise was the first to shew that ligatures of reserve, or ligatures of safety, as they were termed, were, in fact, sources of much mischief and danger, and they have accordingly been long banished from practice, at least in this country.

The well known experiments of Dr. Jones have since demonstrated the pernicious consequences of interposing pads or compresses, between ligatures and the coats of arteries included in them, as well as the advantages of using round, firm ligatures, instead of flat or large ones. To the knowledge derived from these experiments, is unquestionably owing much of the success that has flowed, from the extension of Mr. Hunter's operation, to the external and internal iliac arteries, and other deep-seated trunks, for, had the use of pads and other contrivances of that kind continued to be considered indispensable, either those operations, which have reflected so much glory on our country, must have been deemed impracticable, or, if performed, must have been productive of evil consequences at least as formidable, if not more so, than the diseases for the relief of which they were instituted. Fortunately, however, the advantages attending the use of simple ligatures having been clearly demonstrated, the surgical treatment of aneurism has, in our day, been carried to a degree of perfection that seemed to leave little room to look for further improvement. Well-informed surgeons were not unaware, however, that there still remained one important practical point to be determined, namely, whether in any case of aneurism, where a ligature could not be applied to the artery between the tumour and the heart, so as to prevent the influx of the blood, a cure might not be accomplished by placing a ligature upon the vessel beyond the aneurism, so as to stop the current of the blood through it, and thereby to cause its stagnation and coagulation, and ultimately its absorption.

The case lately published by Mr. Wardrop has decided this question, in a manner alike honorable to the art of surgery and creditable to the surgeon. In two unsuccessful cases only have ligatures been applied beyond the tumours. One of these was a case of femoral aneurism, situated close to the groin, in which Deschamps secured the artery below the tumour; but, if we except the commendable candour with which the facts were recorded, there was nothing, in the conduct of that case, creditable either to the art, or to the operator. The other case was one of aneurism of the external iliac, in which, it being impracticable to secure the artery above the tumour, with any prospect of success, Sir Astley Cooper, with his accustomed zeal, gave the patient a remote chance of recovery by taking the vessel up below the aneurism. Although the patient survived the operation for several weeks, and although at first there seemed reason to hope for a fortunate event, yet, upon the whole, the case held out but little encouragement to surgeons to follow the practice in question, and room was barely left to hope that a more favorable result might be obtained, were the operation to be performed under such favorable circumstances, as were united in Mr. Wardrop's case, and which the skill and firmness combined in that gentleman's character, enabled him at once to appreciate and take advantage of.

We shall now submit to our readers, in the author's own words, his account of the case, with only this remark; that, while we fully concur with some of our contemporaries, who have pronounced this one of the boldest operations on the records of surgery, we regard it as completely exempted from liability to any charge of rashness, by the philosophical grounds on which it was undertaken, and by its successful result.

"A lady, 75 years of age, after a very violent fit of coughing, perceived a swelling on the right side of her neck, a little above the clavicle. When I saw her, eight days afterwards, the tumour had all the characters of an aneurism of the carotid artery, and had become as large as a fist; but was so situated that it was quite impracticable to tie the vessel below the tumour, so closely did it come in contact with the clavicle. The tumour continued to increase in size, and on the eleventh day after it was first observed, it had acquired a formidable aspect, the scapular portion having become very red and painful, the pulsation, which was very strong throughout the whole swelling, being here particularly so, and the parietes feeling extremely thin, and as if ready to burst.

"It was evident that the patient's life was now in the most imminent danger, and in this hopeless condition it forcibly struck me, that it might be highly expedient to tie the carotid artery above the aneurism, in the hope that, by thus stemming the current of blood through the vessel, nature might establish a new channel to carry on the circulation, allow the blood in the tumour to coagulate, and the sac and vessel to contract and be obliterated, as take place after the common operation. There were circumstances which made this case particularly favourable for resorting to such a measure; the aneurism had been of short duration, the patient, though far advanced in years, had a healthy constitution; she was of a tranquil disposition, and eager that something should be done for her relief. Besides, the diseased artery was most favorable for the proposed operation, for as no branches are sent off from the carotid artery until it divides into the external and internal, the process of coagulation would not be interrupted by the continuance of circulation through collateral branches in immediate contiguity with the aneurism. The operation also appeared practicable in this instance, as the aneurism, though large, extended upwards so as still to leave sufficient space for the application of a ligature between the tumour and the division of the artery.

"Under these impressions, and with the approbation of Dr. Veitch and Mr. Glen, who also attended the patient, I undertook the operation, and the result of it has, in my opinion, fully authorised the measure; and I trust that the future experience of others will confirm its utility. The operation consisted in making an incision through the skin and cellular membrane, rather more than an inch and a half in length, commencing it immediately above the tumour, and extending it on the tracheal edge of the mastoid muscle, and in the direction of the carotid artery, taking care to avoid the large superficial veins. The subsequent part of the dissection was chiefly made with a silver knife, guided by the finger, and there was no particular difficulty in reaching the artery but what might have been anticipated, from its great depth, from the necessary limits of the incision, and from the numerous large veins which were carefully to be avoided—particularly a branch which extended across the middle of the incision to the internal jugular, and which consequently diminished the space in which the artery was to be taken up. After a careful dissection, which was tedious from its being necessary to tear the parts with the silver knife, the artery was so completely separated from the adjacent parts, that the point of the finger could be readily passed between the vessel and the vertebræ, and the aneurismal needle, of which I have annexed a particular description,* was passed round the artery with singular facility, taking care to avoid the par vagum which was distinctly felt behind the finger. The vessel being previously ascertained to be healthy, one ligature was tied round it, as close to the tumour as the incision would admit, and the lips of the wound were stitched together by a suture, without any farther dressings. The aneurismal tumour was covered with adhesive plaster, in order to protect the tender skin, and at the same time to keep up a certain degree of pressure.

"I thought it probable that the resistance to the circulation, which the ligature would necessarily occasion, might, for a short while at least, after its application, be followed by an increase in the distention of the tumour; instead of which, however, there was an immediate decrease in its bulk, marked by a considerable corrugation of the skin at the base, as well as a diminution of its redness. The ligature of the artery did not seem to produce any change in the mental functions, or any unnatural feelings in the head; on the contrary, the patient passed the night after the operation more comfortably than that previous to it, the tumour being accompanied with less uneasiness.

"A progressive diminution in the bulk of the aneurism, and in the strength of its pulsations took place, so that on the fourth day after the operation it seemed to have diminished nearly one third in its bulk; the upper and tracheal portions had lost all pulsation, and only the scapular portion retained an obscure undulatory thrill. The integuments, which had lost their redness, now evidently became more inflamed, and during the fifth and sixth days there was a distinct increase in the size of the tumour, and it pulsated more strongly, which seemed partly owing to several severe fits of coughing. This apparently unfavourable change was, however, followed by a decided amendment; and eight days after the operation the swelling again began to diminish, and the pulsation became more obscure, so that on the fourteenth day it was not much larger than half its bulk at the time of the operation, and no pulsation could be detected in any portion of it; merely a slight vibration in some parts which seemed to be produced by the pulsations of the contiguous vessels, which were now enlarged, particularly the inferior thyroid artery.

"The redness of the skin, however, continued to increase and that of the scapular portion of the tumour to become more and more of a purple colour, till, at last, ulceration commenced on the most prominent part. Several considerable-sized portions of coagulated blood were discharged along with some healthy pus through the ulcerated opening; and on the 20th day after the operation, the ulceration of the integuments had closed, and nothing of the tumour remained, but some wrinkling of the skin, and a considerable degree of thickening of those parts on whieh the base of the tumour had rested. These continued to diminish, and at the end of the fifth week, from the time of the operation, the neck had nearly resumed its natural form, a slight degree of inequality only remaining; the ligature had come away, and the patient's general health, to the management of which the greatest care had been bestowed, appeared now to be completely re-established.

* See the Appendix to this Paper.

"This case appears to me to prove satisfactorily, the possibility of the success of this mode of operating for aneurism, and of the important advantages which are likely to be derived from it, more especially in those cases which have hitherto been considered beyond the aid of surgery.

"The operation may also, under particular circumstances, be preferable to tying the ligature between the aneurism and the heart, even in cases where that operation is practicable. For, as in that which I have just narrated, had it even been possible to have tied the artery between the tumour and the heart, how much more dangerous and difficult would the operation have been, and how much greater would have been the risk of secondary hæmorrhage at the place of the ligature.

"The only circumstance which must be considered as indispensable to the success of this mode of operating is, that there be no vessel arising, either from the sac itself, or from the artery between the sac and the ligature, sufficiently large to keep up the circulation of the blood through these parts, and thus prevent its coagulation. I say sufficiently large, for it is perfectly ascertained that, after the common operation of tying the artery between the tumour and the heart, pulsation to a certain degree often continues for some time, notwithstanding which, neither the gradual process of coagulation in the tumour is prevented, nor the subsequent contraction and condensation of the aneurismal sac and insulated portion of the artery.

"When an aneurism is cured spontaneously, it is evident that in general the process must in like manner be slow; and that the circumstance of the circulation through the tumour being rendered languid, equally suffices to admit of the blood coagulating, as if circulation was completely stopped.

"These considerations lead me therefore to hope, that the operation of tying the artery beyond the aneurism, will in many instances be successful, even though the current of blood through it be not completely stemmed. To ensure, however, this being done, the ligature should be made as close to the tumour as possible, in order to preclude the chance of leaving a branch between the tumour and the ligature, which would carry on the circulation; and I can even conceive cases wherein it might be practicable to tie such branch separately, so as effectually to prevent the circulation being carried on through the aneurism." 10.

In the appendix, Mr. Wardrop gives a description, which is accompanied with a plate representing the simple instrument, for which the profession is indebted to the ingenuity of Mr. Bremner, and which appears to us, as Mr. Wardrop found it in this instance, extremely well calculated to aid the surgeon in passing ligatures round arteries, even when deep seated, with the least possible disturbance to the neighbouring parts. For a view of this plate and of one representing the patient after the ligature was applied, we must refer to the original paper.

We have the satisfaction to be enabled to add, that we have learnt, upon enquiry, within a few days of the present (21st January,) that the patient continued without a vestige of the tumour to shew on which side it once existed, and in the enjoyment otherwise of uncommonly good health.

Before quitting this subject, we think it proper to caution the young surgeon not to be led, by the success of this operation, into too hasty an imitation of it. We think it highly probable, that in few other arteries in the body than the carotid, would this operation have succeeded. It has been long ago observed that Nature, or rather disease, performed the very same operation which Mr. Wardrop has so successfully imitated by art. Thus, Sir Astley Cooper has mentioned an instance (*Med. Chir. Trans. V.* 1, *p.* 12,) where the common carotid was obliterated by the pressure of an aneurism of the aorta, which extended up the neck; but what is more to the purpose, Mr. Hodgson met with a case, where there was a small aneurism at the origin of the subclavian artery, cured spontaneously by the deposition of lamellated coagulum, the consequence of an obliteration of the artery *beyond* the aneurism, by the pressure of an aortic aneurism. Thus one aneurism cured another, serving, in fact, as a ligature. A great many years ago, in the first volume of Transactions of a Society for the Improvement of Chirurgical Knowledge, Sir Everard Home remarked that sometimes, "the sac is protruded along the outside of the artery, and, by its pressure upon it, obliterates, in many instances, the lower orifice which communicates with the artery, and produces a total stagnation of the blood in the sac." Now the only chance which such an operation offers, depends on there being no branches originating from the sac or from the artery between the ligature and the tumour—and it is evident that, of all arteries, the carotid presents the best field for such an attempt. For very obvious reasons, the two attempts that were made by tying the artery in the groin, had little chance of success, on account of the vessels going off in that neighbourhood; whereas, if a ligature be placed beyond an aneurism of the common carotid, it is next to impossible, that there can be any conduit for a stream of blood through the sac. "There is strong reason," says Mr. Hodgson, "to believe, that if no branch originated from the aneurism, or from the artery below (beyond) the aneurism, the blood would coagulate in the tumour, and a cure would be accomplished by the absorption of this coagulum and the subsequent contraction of the sac."—*On the Arteries, p.* 302.

Mr. Wardrop then has the honour of first verifying, by a successful operation, the speculation of former writers.

---

20. *Staphyloraphia.** In certain cases, and those of not very rare occurrence, there is a division of the velum pendulum palati, or soft palate, as a congenital defect, somewhat analogous to the harelip. This defect is not merely a deformity like the latter, but is a serious impediment to the functions of deglutition and speech. This misfortune has been, till lately, considered as irremediable; but within the last five or six years, a number of individuals have been cured by an operation, which M. Roux appears to have been the first, or among the first to perform—and that on a young medical gentleman, Mr. Stephenson, from Canada. This gentleman was born with a complete division of the velum palati, through its centre, where a triangular space was left instead of the uvula. In the early periods of life Mr. S. was fed with much difficulty. Afterwards other inconveniences were felt. When he vomited, the matters ejected from the stomach passed through the nostrils, as well as liquids, when he attempted to swallow them without

* M. Roux. Archives Générales, Avril, 1825.

holding back the head. He could not blow air into any instrument through the mouth, and his voice and pronunciation were greatly deteriorated and embarrassed. On looking into his mouth, M. Roux observed certain momentary contractions of the parts, by which they were brought nearly in apposition. He was surprised at this power in the muscles of the palate, and he immediately conceived the idea of a reunion of the divided portions, on the same principle as the operation for the hare-lip. The means to be employed were evidently excision of the edges, and then retention of them in contact, by suture. The suture employed then and since has been the interrupted. Contrary to the procedure in the hare-lip operation, M. Roux determined to place the ligatures *before* he incised the edges of the cleft palate, so that when this last was done, he should have only to draw and tie the threads, and thus finish the operation. For our own parts, we should have thought the presence of the threads would have embarrassed the operation of excision; but M. R. thinks otherwise. Small crooked needles were employed to pass the threads, and a probe-pointed bistoury, with crooked forceps were the instruments for excision. Three ligatures were placed—one near the bottom of the division, one in the middle —and one near the extremity. The operation lasted fifty minutes, and when finished, Mr. Stephenson's voice was found to be quite altered, and his pronunciation rendered natural. Strict silence and rigid abstinence were then enjoined, and the case went on without any serious accident. Two of the ligatures were cut away on the evening of the third day. The third was suffered to remain 24 hours longer. No food was permitted to be taken all this time, and only a few spoonfuls of soup now. Silence was enjoined till the 8th day. The point of the uvula was observed to be bifid, and a piece was cut off the longer division. Mr. Stephenson's voice, though amazingly improved and improving, was still somewhat nasal, at the end of six months from the operation.

This congenital deficiency is found in different degrees, but by far the most common form is that which Mr. Stephenson presented. The division, in all cases, is found to be precisely in the middle of the uvula and velum pendulum palati. Seven cases are detailed of this operation, where there was simple division of the soft palate, and no separation of the bones. The eighth, ninth, tenth, and some other cases, in which there was separation of the bones, as well as of the soft palate, were unsuccessful. In the 13th case, where there was a partial separation of the bony palate, namely in one third of its posterior part, and to the extent of five lines, our author had more success. A partial union of the soft palate took place, not at its anterior but posterior part, by which result an aperture was left, just above the uvula, which appendix was formed by the partial union.

Such are the facts which M. Roux has brought forward, and we think they are such as to encourage expert surgeons to exercise their dexterity in relieving such infirmities wherever they are met with.

21. *Hyoscyamus and Camphor in Gonorrhœa.** Mr. Bell is by no means satisfied with the plans which have been recommended for the cure of gonorrhœa, especially in its chronic stage. He thinks, and probably with reason, that "the disease, in most cases, would exhaust itself more rapidly, if not interfered with, than it is observed to do, when treated in the common method." Perhaps gonorrhœa is not the only disease to which this observation might apply—but this is delicate ground to tread on, now that the newspapers are becoming the vehicles for medical discussions. Gonorrhœa is, according to Mr. Bell, "a *constitutional* affection of the *irritable class*, and should therefore, from that circumstance, be treated *throughout all its stages*, on those principles which have for their object the allaying of irritability." We cannot help demurring to this sweeping position of Mr. Bell, that gonorrhœa is "a *constitutional* affection of the irritable class." The disease may indeed occur in *constitutions* of the irritable class, but it is a strange confusion of ideas to designate it, on that account, a *constitutional* affection. It is assuredly a *local* disease, capable, like almost all other local diseases, of producing constitutional disorders. This indeed is made out even by Mr. Bell himself.

"Every practitioner must be aware, that, even after the local acute inflammatory symptoms of a mucous membrane have been allayed, a degree of subacute irritation, attended with more or less turgescence of the vessels of the part, remain, which are also accompanied by general derangement in the functions of the digestive organs. On taking this view of the subject, the impropriety of employing those drugs, which tend either to disorder, by their general stimulating influence, or by their local specific effects, the functions of digestion, is apparent,—for it is well known, that a very intimate sympathy prevails among all the mucous surfaces." 76.

Our author, proceeding on this view of the case, considers it necessary, after the acute symptoms are subdued, to proceed on the plan of allaying irritation, both local and general. To this we have no objection, provided the means of allaying irritation will remove the gonorrhœa, for it is too late in the day to speculate on the nature of a disease without direct reference to the treatment. Mr. Bell thinks it is very difficult to determine when the inflammatory stage of gonorrhœa has subsided, since the absence of pain, ardor urinæ, and chordée does not afford sufficient proof. It frequently happens, he remarks, that after the disappearance of these symptoms, a degree of uneasiness remains, which is more difficult to remove than the primary symptoms of the complaint. This uneasiness is characterised by painful spasms after micturition, irritability of the erectile tissue of the penis and cervix vesicæ, and tenesmus, arising from irregular action of the sphincter and levator ani. Under such circumstances the impropriety of pursuing the stimulating plan he thinks is evident.

"In this state of the organs, a single dose of copaiba, cubeb pepper,

* Mr. Benj. Bell. Ed. Journ. Med. Science, No. I. Jan. 1826.

or tincture of cantharides, will bring on an attack of hernia humoralis, cystitis, inflammation of the prostate, or spasm of the neck of the bladder, so violent, as to occasion retention of urine. I have not drawn an exaggerated picture; such events are frequent, and will be so until the stimulating diuretics, and, indeed, diuretics of every description, are excluded from practice in the treatment of gonorrhœa. Even *simple diluents* do not appear to me to be salutary, at least in the quantity in which they are usually prescribed. They load and upset the stomach, fatigue the kidneys, and irritate the bladder, by calling it too frequently into action.

"Although *diuretics* and the *stimulating balsams* should not, in my humble opinion, be resorted to in the treatment of gonorrhœa, or of that *numerous class* of diseases ranged under the general appellation of *gleet:* still I do think that, in some of the *sequelæ* of gonorrhœa, they may be employed with advantage. In that state of debility of the bladder, or of its cervix, which occasionally follows an inveterate or ill treated gonorrhœa, when the organs do not contract to a sufficient extent, or contract irregularly, they are frequently serviceable.

"There is another state, also, in which they may be employed with some prospect of advantage, and that is, when there appears to exist a state of partial or total atony of the erectile tissue of the penis, accompanied by a thin, watery, mucous discharge.

"This state is particularly annoying to the patient, not from the pain attending it, as that is generally very trifling, but from the impossibility of his procuring an erection:—he is, in truth, impotent. It is the impotency of the debauchee, which has affected him—an impotency, in some cases at least, incurable, as it seems to depend occasionally upon a varicose state of the veins which convey the blood from the cavernous and spongy structures of the urethra." 78.

Our author divides gonorrhœa into four distinct periods—"each differing *essentially* from the other," and each requiring a different treatment. In all of these stages, however, he avers that there is a common feature—*constitutional* as well as local irritability. We shall give the author an opportunity of explaining his own doctrines and practice.

"*First*, or *Acute Irritable Stage.*—During this period of gonorrhœa, when inflammation has not yet taken place, every effort should be made to lower the system; for it has been observed, that the severity of the inflammatory stage depends in some measure upon the degree of irritation which has previously existed.

"In order to accomplish this object, the patient must be confined to a low diet, his bowels should be relaxed by means of mild purgatives, and the cuticular secretion promoted by the daily employment of the general warm bath. The purgatives which seem best suited for allaying mucous irritation, and more especially where the urinary organs are the parts principally concerned, are those of that class which promote free and watery alvine evacuations, without acting violently on the colon or rectum, or affecting the chemical composition of the urine,

Sulphur alone, or combined with magnesia, senna, castor-oil, blue pill combined with ipecacuan, or calomel in conjunction with antimony and opium, are peculiarly applicable, as they have a secondary effect upon the skin. Rhubarb, the gum-resins, and neutral salts, should never be prescribed.

"*Second*, or *Inflammatory Stage*.—So soon as inflammatory action is fairly established in the mucous membrane of the urethra, recourse must be had to more active treatment. In plethoric habits, blood may be taken freely from the arm; and, in more delicate constitutions, cupping of the loins will be found advantageous. Leeches I am no friend to in gonorrhœa, as they irritate and annoy the patient, and in some cases give rise to troublesome erysipelas, or œdema; and, when applied to the perineum, are apt, from the irritation they produce, to occasion painful erections, and to interfere with the use of fomentations, which are in every instance valuable adjuncts. I am not sure how far the general warm-bath is useful in the acute inflammatory stage, as in several cases it has seemed to exacerbate the symptoms.

"It is in this stage that ardor urinæ, nocturnal erections, and chordée, exist; and I have seen the agony attendant upon the latter symptom so severe, that the patient contemplated the hour of bed-time with a degree of horror, bordering on despair. In the case of a West Indian gentleman, the chordée was so dreadful, that, at his own hand, he nearly committed suicide, by taking an overdose of opium and digitalis.

"Anxious to discover a remedy which might tend to alleviate the agonies of chordée, without disordering the digestive organs, as opium generally does, it occurred to me, that, as camphor is sometimes used with great benefit in irritable affections of the bladder, and aware of the experiments of Chrestien on the subject, I determined to give it a fair trial. My experiments with regard to this drug have been hitherto attended with success.

"It must be observed, that camphor cannot be exhibited alone, as it is very apt to give rise to nausea. In combination with hyoscyamus, however, that objection is obviated, for the compound has never, so far as I have observed, given rise to any gastric uneasiness.

"When combined, in the form of a pill, with hyoscyamus, camphor may be pushed to a very considerable extent; and in more than one instance, when severe chordée, attended with spasm of the cervix vesicæ existed, I have prescribed, in the course of twenty-four hours, one drachm of the latter, in combination with two scruples of the former. In general, half the above quantity will be found sufficient; and the rule to be observed in giving them is very simple, for, on the occurrence of the slightest symptoms of vertigo, their exhibition should be suspended.

"Camphor and hyoscyamus possess several advantages over other narcotics; they appear to have a decided effect in diminishing the force of the circulation, and they allay irritation, and do not occasion costiveness, or a diminution of the secretion of mucus from the lining membrane of the intestines. In truth, they do not interfere with the exhibition of other medicines.

"Camphor may be employed with advantage in all the stages of gonorrhœa; and in those rare cases where it is found not to agree with the stomach, it may be used in the form of a liniment, rubbed five or six times a day upon the loins, perineum, or groins.

"*Third*, or *Chronic Inflammatory Stage.*—It is at this period of gonorrhœa, when all the acute symptoms have disappeared, that the sagacity of the practitioner has full scope. It is the stage of difficulty. Depleting remedies will be found to prove noxious, and stimulants dangerous. A purgative may induce a serious relapse; and one dose of copaiba, cubebs, tincture of cantharides, or a single astringent injection, give rise to hernia humoralis, cystitis, or catarrhus vesicæ.

"Quiescence will, in this stage, be found the most valuable remedy; the cold hip-bath may be used, and nauseous gruels and slops may be relinquished for animal food. A mild alterative course of the blue pill, with antimony, or ipecacuanha, will also, in many cases, be attended with advantages; and an occasional dose of magnesia, with colomba or gentian, will be of service.

"*Fourth*, or *Chronic Irritable Stage.*—We have now arrived at that stage when the lips of the urethra have assumed their natural appearance, when almost all discharge has ceased, and when the patient experiences merely an occasional slight uneasiness during micturition. This state of atonic irritability, if I may be allowed the expression, is sometimes very obstinate, and requires particular attention on the part of the surgeon. It will yield in many cases to local or general cold bathing and tonics; and in this state I have observed the sulphate of quinine to prove of great service." 80.

Our own experience on this complaint, which has not been very limited, inclines us to think that Mr. Bell leans too much to depletion, and is too much afraid of what he calls *stimulation*. But it is one thing to drink brandy, and another to take copaiba or cubebs. No man, who has attentively watched the effects of medicines in gonorrhœa, will confound the two latter medicines under the general head of stimulants. Their usual effects are diminution rather than increase of irritation in the urinary organs—and therefore we suspect that Mr. Bell draws a little on theory and overlooks some practical facts on the foregoing observations. Still there are some good remarks made, and some judicious rules laid down by the author, wherefore we recommend them to the attention of our readers.

---

22. *Hæmorrhage in Lithotomy.** In the operation for stone, more than in any other surgical operation, an unusual distribution of the arteries is productive of great embarrassment to the surgeon and danger to the patient. No skill or dexterity, in such a case, can always avail; for the divided vessel is often beyond the reach of ligature or pressure.

* Mr. John Shaw. Med. and Phys. Journal, Jan. 1826.

Unfortunately there are never wanting people in our profession, ready to seize on such an accident, for the purpose of gratifying a diabolical propensity to defame and traduce the character of those who may be more eminent or distinguished than themselves. A Cooper, a Brodie, and a Bell have, in this way, been maligned by the most ignorant and contemptible of the profession! The following passage in the 9th volume of Baron Boyer's great Chirurgical work deserves to be transcribed here. "Hæmorrhage, says he, is one of the most common accidents of lithotomy, and is often placed to the account of the operator, or of the mode of operating, but almost always unjustly;—for the vessels of the part divided present so much variety in their situation and direction, that the most expert surgeon (chirurgeon le plus habile) is not always able to avoid them, whatever precaution he may take." p. 429.

We all know the outcry that was made about an unsuccessful operation lately performed by Mr. Shaw at the Middlesex Hospital, and the lies and misrepresentations which were published on that occasion. Mr. Shaw has properly laid the case before the public, with a drawing, in which the unusual distribution of a branch of the Ischiatic or Pudic traversing the neck of the bladder, and lying directly in the way of the incision is clearly demonstrated. From this vessel a great hæmorrhage took place, and caused the death of the patient. The case itself is well deserving of record, and is probably of more frequent occurrence than is generally supposed. We shall give the particulars in this place.

The patient was a stout countryman, who came into the hospital, without knowing what was his complaint. On sounding, the stone was supposed to be small, and attempts were made, but without success, to extract it per urethram.—At length the operation was performed. On feeling the staff through the face of the prostate, Mr. Shaw cut upon it, and carried his knife forward through the membranous part of the urethra and the prostate gland. On making this incision there was a gush of blood, but the operation was prosecuted, and two small calculi were readily extracted, in the course of three or four minutes. The blood flowed profusely, and Mr. S. feared that he had cut the pudic, but finding it beat strongly and distinctly under his finger, he was relieved from that apprehension. Pressure on that vessel had no effect in stopping the hæmorrhage. The patient was kept on the table for some time, and the flow of blood appearing to lessen, he was put to bed, while cold applications were made to the lower part of the body. The bleeding, however, was renewed, and the wound was exposed to a strong light, when the pudic was distinctly seen beating all along the ramus. On sponging the wound clean, the blood was seen to ooze apparently from the bladder. The depth of the place whence it issued (4½ inches) prevented the possibility of placing a ligature on the vessel. A piece of sponge and some lint were wrapped around a canula, which was left in the bladder. There was very little more hæmorrhage; but the patient soon became restless—complained of much pain in the chest and abdomen—was ultimately affected with violent spasms, and died at 11 o'clock on the same night. On the following morning the vessels of the pelvis were carefully injected, and the unusual distribution to which we before alluded became manifest.

From an extract and a drawing copied from the great work of Tiedman, the actual variety in question is clearly described by that celebrated anatomist. Winslow also describes the artery of the penis as not unusually passing along the prostate gland. Mr. Harrison, the able demonstrator of anatomy in Dublin, and author of a very excellent work on the arteries, mentions his having occasionally observed the pudic artery, on one or both sides, to be very small, while the internal iliac was found to have given off a distinct branch running along the side of the bladder and prostate gland, and passing beneath the arch of the pubes with the dorsal veins, becoming the dorsal artery of the penis. "*Should such a variety, says he, exist in one who was to become the subject of the lateral operation of lithotomy, I fear this artery must be wounded.*" This prediction was unfortunately verified in Mr. Shaw's case—but he who could be so diabolically inclined as to trumpet forth the accident in the form of a charge of unskilfulness against this meritorious young surgeon, and distinguished anatomist,

"Is fit for treason, stratagems, and spoils!
"The motions of his mind are dull as night,
"And his affections black as Erebus—
"Let no such man be trusted."

---

23. *Fracture of the Neck, and upper part of the Femur.** Mr. Guthrie considers the interesting publications of Sir Astley Cooper and the discussions to which they led, as having, by no means, exhausted the subject, nor given to it all that elucidation which it deserves. Fractures and dislocations of the parts abovementioned are often, with difficulty, ascertained, in consequence of the depth at which the head and neck of the bone lie—hence their displacement, together with its particular nature, is sometimes more satisfactorily ascertained by certain attendant and even distant signs, than by the closest investigation of the part itself.

The great difficulty hitherto has been to distinguish between dislocation of the head and fracture of the neck of the femur. Certain diagnostic marks indeed are laid down for each, but, as Mr. Guthrie conceives that they are common to both, they require revision collectively rather than separately. We shall give Mr. Guthrie's own words.

"The dislocations hitherto demonstrated, which may be mistaken for fracture, are of two kinds:

"1. Upwards and backwards, on the dorsum of the ilium.

"2. Backwards, into the ischiatic notch.

"In the first kind of dislocation, or upwards and backwards, the head of the femur is removed from the acetabulum, drawn upwards, and turned

---

* Mr. Guthrie. Med. Chir. Trans. Vol. xiii, part I.

backwards on the dorsum ilii. The trochanter must, consequently, be forwards, and nearer to the anterior superior spinous process of the ilium. The limb is necessarily shortened, from one inch and a half to two inches and a half. The knee is turned inwards, and a little advanced, and the great toe rests on the instep of the other foot. The head of the bone is firmly fixed in its new situation, the limb cannot be drawn outwards, or fully separated from the other, although it admits of motion inwards, when the head of the bone may be felt in most cases moving on the dorsum ilii. The trochanter major is less prominent, the hip will, consequently, be flatter, and its rotundity will be diminished. There will generally be a greater degree of distortion than is apparent in any other kind of injury.

"In the second kind of dislocation, or backwards into the ischiatic notch,* the limb is from about half an inch to one inch shorter than the other, but generally not more than half an inch. The trochanter major is behind its usual place, but is still remaining nearly at right angles with the ilium, with a slight inclination towards the acetabulum. The head of the bone is so buried in the ischiatic notch that it cannot be distinctly felt, except in thin persons, and then only by rolling the thigh-bone forwards so far as the comparatively fixed state of the limb will allow. The knee and foot are turned inwards, but less than in the dislocation upwards, and the toe rests against the ball of the great toe of the other foot. When the patient is standing, the toe touches the ground, but the heel does not quite reach it. The knee is not so much advanced as in the dislocation upwards, but is still brought a little more forwards than the other, and is slightly bent. The limb is so fixed that flexion and rotation are, in a great degree, prevented.

"There is a third kind of dislocation mentioned, but which has not been demonstrated. The dislocation is upwards, but, contrary to what takes place in the first species, the head of the bone is turned forwards, the trochanter backwards. When such an accident occurs, the knee and great toe must be very much turned outwards, the limb shortened from an inch and a half to two inches and a half, and fixed so as not to admit of rotation inwards. The head of the bone will be distinguishable on the dorsum of the ilium, the trochanter will be deeply buried, and turned backwards. The hip must be greatly flattened, constituting, altogether, a diagnosis not easily mistaken, whenever such a case occurs." 106.

Fractures of the cervix may be confounded with dislocation of the os femoris upon the dorsum ilii, and with that into the ischiatic notch, as in both these dislocations the limb is shortened. The eversion of the foot has been considered diagnostic of fracture, together with mobility of the limb. The eversion, however, is not constant—nay *inversion* takes place sometimes, as noticed by Paré, and said by Desault to occur in the proportion of one case in four, which is oftener than modern surgeons admit. But even when the eversion does occur, some hours must elapse after the accident, before it assumes its most decisive character, as the muscles require time for determined contraction.

Mr. Guthrie had conceived, and taught with others, that when the foot was turned in—or neither in nor out, the fracture must be in the cervix of the bone, near the head, "so that the portion attached to the

* Sir A. Cooper on Dislocations, &c. page 66. 3d edition.

trochanter passed behind that remaining with the head of the bone, in this manner causing the turning inwards of the great toe—or that, the capsular ligament being torn, it passed backwards behind the acetabulum, giving rise to the same appearance." This explanation Mr. Guthrie has proved to be unsatisfactory. We shall now introduce a couple of cases, which will have more weight than hypothetical explanations.

"In January, 1823, I visited a lady who, seven days previously, had slipped at the entrance of her house, and fallen on the left hip. She suffered great pain in the part, at the inside of the thigh, and in the course of the sciatic nerve. She was lying across her bed with the affected limb supported by a stool, the toe and foot being very much everted, and the heel resting below the ancle of the opposite side. On placing her in the extended position, the limb seemed little shorter than the other, was moveable in every direction as far as it could be tried, on account of the extreme pain it occasioned. The upper part of the thigh or hip was swelled, and a derangement of the trochanter could be distinguished, but the nature of it could not be distinctly ascertained; a crepitus was not discoverable. The injury was declared to be a fracture exterior to the capsular ligament, but, after the first day of treatment, the great toe turned inwards, resting against the toe of the opposite side, and continued to do so for several weeks, giving rise to a great deal of annoyance on my part, and several subsequent examinations, from the fear of having misunderstood what appeared at first to be a well-marked case. This lady can now, after an interval of eighteen months, walk with the help of two sticks, the limb is little shortened, and the foot is in its natural position.

"Sarah Gibson, aged 90, fell, on the 9th of January, from a high stool on which she was sitting, upon the left hip, and being a heavy woman suffered considerable injury. I saw her two days afterwards with Mr. Dillon of Judd Street, and found the marks of a considerable contusion having been sustained by the part, which was very painful and swelled. The limb was rather more than half an inch shorter than the other, and the great toe turned inwards, as in the preceding case, in a manner sufficiently marked, although not quite so decidedly as in a case of dislocation. The limb was moveable in every direction, but these motions were attended by considerable pain, and it could be easily extended to the same length as the other. No crepitus could be distinguished. The patient died on the 22d February, forty-four days after the accident, and on dissection a fracture was discovered external to the capsular ligament. The little trochanter was broken off, and with it the attachment of the psoas and iliacus muscles. The head and neck of the femur were separated from the shaft by a diagonal fracture, extending from the upper and outer part of the trochanter major to the trochanter minor, so as to leave the insertions of the pyriformis, gemelli, obturator externus and internus, and quadratus, with the head and neck of the bone. The glutæus medius formed a bond of union at the upper part of the trochanter major, between the broken pieces, retaining them in contact. The capsular ligament was not injured, and no steps whatever appeared to have been commenced to repair the mischief which had been committed. The great age of this person, and the trifling nature of the accident giving rise to so serious a fracture external to the capsular ligament, deserve remark." 111.

Mr. Guthrie remarks that, in the dead body, and when muscular power is extinct, the toes turn outwards, from the weight of the foot and limb preponderating in that direction. If the living body be placed on the back, in the horizontal position, but in a state of quiescence, the same thing takes place, the weight of the limb being greatly assisted by the rotators of the thigh outwards, viz.—by the pyriformis, the gemelli, the two obturators, and the quadratus. "Any kind of fracture, admitting of displacement, within the insertion of these muscles, will have the effect of increasing this eversion, as it diminishes the resistance to the contractile power of these muscles, which, stimulated to action by the injury, readily overcomes that of their antagonists, and particularly of the tensor vaginæ femoris, the gluteus minimus, and part of the gluteus medius." Mr. Guthrie makes a number of ingenious remarks, indicative of his perfect knowledge of the anatomy and functions of the many muscles and ligaments about the hip joint, and then draws the following inferences:—

"1st. That whilst the eversion of the foot is characteristic of fracture, its absence does not indicate the non-existence of fracture.

"2d. That the inversion of the foot is equally characteristic of fracture as of dislocation, and is only distinguishable, with reference to these two different states, by comparison, or a due estimate of the degree of inversion.

"In the dislocation upwards and forwards, or on the dorsum ilii, the inversion of the foot is complete, the great toe is turned inwards, and rests on the instep of the opposite foot, being the first or greatest degree of inversion. The limb is generally two inches shorter than the other.

"In the dislocation backwards into the ischiatic notch, the inversion of the foot is decidedly marked, but is not so complete as in the preceding case. The knee and great toe turn in, the latter resting against the ball of the great toe of the opposite foot, and not admitting of rotation outwards. The limb is but little shortened, yet cannot be lengthened without great force.

"In fracture, the inversion of the foot is less complete, the great toe merely turns to the opposite one, and sometimes scarcely does this; the limb is but little shortened, is easily everted, readily moved in almost every direction, although not without pain, and is restored to its proper length on the application of a very moderate extension. This constitutes the third degree of inversion.

"3. That inversion of the foot does not take place in fracture within the capsular ligament, and that this symptom is rather diagnostic of a fracture through the trochanter major, a portion of it being continuous with the shaft of the bone." 115.

The shortening of the limb, both in dislocations and fractures, has given rise to much discussion—a circumstance, Mr. Guthrie thinks, attributable to the not distinguishing between the symptoms which may be called *immediate*, and those which are consecutive—also, from not considering the positive nature of the accident itself, which sometimes does not allow of any displacement of parts, several instances of which are on record. Mr. Guthrie has known a man walk several steps after a fall on the trochanter, then become incapable of moving the limb, although no retraction of it took place—yet, on examination, several weeks afterwards, the deposition of bone behind the trochanter, uniting the parts, could be readily perceived. Three or four hours, however, must elapse before retraction or eversion assumes its most decisive character. The shortening of the limb, in recent cases of fracture of the cervix, will rarely exceed an inch and a half—and seldom, he thinks, will even be so much. "A diminution of the length of the limb to the extent of three or four inches, cannot, I presume, take place in a case of fracture within the capsular ligament, unless the ligament be torn through, and then only as the consequence of a long continued and unopposed action of the muscles, combined with the weight of the body on the limb." The greater or less extent of shortening of the limb, considered separately, cannot form a criterion as to the immediate seat of injury. As a general rule, however, Mr. Guthrie would say, "the shortening of the limb within the first 48 hours, is less when the fracture is external to the ligament, than when it is within it." A greater degree of pain, swelling, soreness, and contusion, would seem to indicate, in general, a fracture external to the ligament, rather than within it. "A crepitus cannot always be distinguished, even when the limb has been drawn to its proper length." Lisfranc avers that the crepitus will always be heard through the stethoscope, if it exist at all; and we think this very probable. Indeed, we have often wondered how surgeons could expect to hear this said crepitus with their ears one or two feet from the limb. If the ear be brought in contact with the integuments over the fracture, the grating will be heard just as distinctly as through the stethoscope. The cylinder can have no power of *increasing* sound; it can merely supply the place of actual contact of the auditory apparatus and the part whence the sound issues. "In a case of *fracture*, motion *can* be fully accomplished in every direction, although it is sometimes attended by great pain. In dislocation, motion cannot be given to the limb in every direction, to the same extent, and least of all, towards complete abduction."

"The shortened limb in dislocation cannot be restored to its proper length without the application of the force of several persons; in fracture it may be readily accomplished by the surgeon alone. In dislocation, the limb once restored to its proper length, remains so; in fracture, it is very soon retracted or shortened as much as before." 120.

We conceive that the surgeon will be much interested in the able and ingenious remarks contained in Mr. Guthrie's paper.

---

24. *Fungus of the Uterus extirpated by Ligature.** Madame C. aged 60 years, of good constitution and rather embonpoint, had borne six children, and had always had severe labours. From the time of her second accouchement, at 22 years of age, she laboured under a descent of the womb, which gradually increased, and at length produced considerable inconvenience. At the age of 45 the menses had become very profuse, and two years afterwards they ceased altogether, but were

* Professor Recamier. Revue Med. Dec. 1825.

succeeded by occasional hæmorrhages. At 50, she became affected with a leucorheal discharge, slightly tinged with blood—at 55, the discharge was dark-coloured and fetid—at 59, the discharge was very profuse, and so fetid as to be unbearable by those about the patient. Meantime she felt the descent of the womb (as she supposed) become greater and greater, till it began to project through the os externum, but without any lancinating or other pain in the part. She consulted M. Recamier and M. Marjolin in October, 1825, and an operation was fixed for the 14th of that month.

On bearing down, a tumour presented itself, and could be drawn out, so as to bring the uterus, to which it was attached, in view. This tumour was cylindrical, three inches in length, an inch and a half in diameter, irregular and ulcerated over its whole surface—of a brownish colour, and covered with a fetid matter. It was very painful to the touch. Its base, which was very hard, was implanted into the inferior part of the womb, but without any neck, or lessening of size. The uterus itself was enlarged and indurated at that part. No cervix uteri or os tincæ could be discovered. That portion of vagina covering the inferior third of the uterus was covered with ulcerations—all the rest of it was healthy. A sound introduced into the bladder could be felt through the rectum, which proved that there was no other tumour or disease than what appeared in view. A needle, with two strong ligatures, was passed through the tumour close to the uterus, and then each ligature tied, by means of a canula, over its own half of the tumour. Considerable pain was experienced when the ligatures were drawn tight. Fomentations were applied to the abdomen—and an opiate was exhibited. Three hours after the operation the pain continued—some vomiting occurred—pulse small—cold sweats. The ligatures were a little relaxed, and all the bad symptoms ceased. But in an hour they returned again, and the ligatures were completely loosened. Thirty leeches to the hypogastrium. The pains entirely went off. At ten in the evening the ligatures were drawn a little. The pain was at first acute, but soon subsided. The patient slept five hours. 15th, The uterus and also the tumour are swelled a little. M. Recamier threw another ligature round the neck of the tumour above the others. To abbreviate the detail, we need only state that each day the ligatures were drawn a little, followed by pain, which generally soon subsided. On the 27th October, (13th day from the operation) the tumour was found to be enlarged to the size of a man's fist, red, and covered with false membranes. 28th, The tumour was completely black. 30th, It was becoming putrid, and sent forth a fetid odour, in spite of the chloruret of lime. It was now cut away near the ligatures. The peduncle of the tumour, together with the ligatures and the canulæ, did not come away till the 11th November, the 29th day from their application. From this time the patient went on rapidly improving, and recovered perfectly.

25. *Extirpation of the Parotid Gland.** Several years ago, we contended that this gland *had been* extirpated, and might be extirpated by an expert surgeon, although the current of surgical authority was decidedly against us at that time. Since then (1817) several authentic cases of this operation have been published—and Sir Astley Cooper, in a letter to Mr. Kirby of Dublin, avers that he twice removed the parotid gland in one year. It is true that, when we view the natural relation of the parotid gland to the important organs in its vicinity, we would be led to side with those who proclaim that any operation for the removal of the gland must be inevitably fatal. But observation shews that those parts are pushed from their natural position by the morbid growth, rather than involved in its structure—and that a layer of cellular membrane continues for a long time interposed between the diseased mass and the important organs to which allusion is made. " The same security," says Mr. K. " afforded by the presence of this substance in the commencing stages of the operation, is derived from it when the tumour is to be disengaged from its deepest connexions. It then gives way before the finger which, as it were, roots behind the mass—or it is broken up by the handle of the scalpel, the blade being unnecessary in the advanced stages of the operation, except for the division of such bands of fascia as the finger may encounter."

The patient on whom Mr. Kirby operated, was a poor woman, about 40 years of age, who had a tumour extending from above the zygoma downwards on the neck, two inches below the angle of the jaw-bone, stretching as far forward on the face as the anterior margin of the masseter muscle, forcing the ear backwards, and raising it outwards from its natural position. It was raised above the surface about the size of a goose-egg—immoveable—painful when handled—surface irregular—integuments of a deep livid colour over the prominent points—pains of a lancinating character, extending over the face, head, and neck, producing sickness and want of sleep.

It would be useless to describe the various steps of the operation, since each case must necessarily require its own mode of extirpation. Suffice it to say, that, after making a crucial incision through the integuments, and exposing the dense and expanded fascia of the gland, the knife and the finger went to work to root out this mass of disease, and at length succeeded, but not without difficulty, and even some danger. The facial branches of the portio dura were unavoidably cut; but the most embarrassing circumstance was a copious hæmorrhage, which occurred from the bursting of a portion of the tumour, while Mr. Kirby was rooting it out from between the pterygoid muscles. The bleeding was restrained by the finger of an assistant, and the extirpation was completed, no perceptible portion of the diseased gland being suffered to remain.—" The space between the pterygoid muscles was void—the auditory tube was fully exposed—the articular capsule of the jaw was brought into view—the finger could trace the length of the styloid pro-

* Mr. Kirby, on Hæmorrhoidal Excrescences.

cess, and on sponging the wound of its blood, it could be seen by those who surrounded the chair." The wound quickly filled with blood when the pressure of the finger was removed; a sponge was, therefore, firmly lodged at the bottom, and all was secured by pads of lint laid as compresses, and by a double-headed roller.

The poor woman was greatly exhausted, but slept the first night tolerably well, complaining only of thirst and inability to swallow. On the second day there was tumefaction, with redness, and fever. On the third day, the tumidity of the face had greatly increased—and there was a diffusion of erysipelatous inflammation over the neck—pulse small and frequent—lethargic. Fourth day, in the same state—discharge fetid, but inclining to suppuration. From this time she went on favourably, and entirely recovered, without any regeneration of the tumour.

The extirpation of the parotid gland must be considered as *one* of the most difficult (if not *the* most difficult) operations in surgery. Mr. Kirby has reason, therefore, to be proud of such a performance, especially when successful, as was here the case.

---

26. *Wound of the Abdomen—Protrusion of the Stomach.** The first of these cases is related by Mr. Benjamin Travers. The subject of it was a female, aged 53, and the mother of nineteen children, who, in a fit of despondency, inflicted on herself a wound in the abdomen three inches in length, extending in a transverse direction, below the umbilicus, and entirely through the abdominal parietes. Six hours afterwards she was admitted into St. Thomas's Hospital, with the greater part of the large curvature of the stomach, the arch of the colon, and the entire large omentum, protruded and strangulated in the wound. On examination, the omentum was found to be detached from the stomach to some extent, and two wounds appeared on the last-mentioned viscus—one a peritoneal graze, half an inch in length—the other a perforation of its coats admitting the head of a large probe, from which a considerable quantity of mucus was observed to issue. The patient was very faint and exhausted when received—pulse 102, and irregular, disposition to hiccup, but little pain in the abdomen. By enlarging the wound, the protruded viscera were, with much difficulty, replaced, a silk ligature having been first placed round the small puncture in the stomach. The external wound was then closed by the quill suture. Warm fomentations were applied, and the strictest abstinence enjoined. *2d day*, She had been sick in the night, the nurse having given her drink, contrary to orders. She is now free from pain—pulse 120, full, and soft—skin warm—countenance improved. Has pain on pressure of the abdomen. An enema ordered. In the evening a dose of castor oil, and 20 leeches to the abdomen. *3d day*, There was this day a considerable exacerbation of fever, and 18 ounces of blood were taken

* Mr. Travers. Ed. Journ. Med. Science, No. I. January, 1826.—Mr. Dix, Med. and Phys. Journal, December, 1825.

from the arm, and twenty more leeches to the abdomen. These means produced relief of the pain and fever, but the bowels were not yet opened. *4th day*, Has had two evacuations—pulse 98, full and soft—considerable tension of the abdomen—three more evacuations in the course of the day. *5th day*, Removed the sutures—wound united, saving at its right extremity, whence a serous fluid exudes in considerable quantity. *6th day*, Craves for food, and is allowed it. She perfectly recovered, and was discharged cured on the 23d December, about two months after the accident.

Mr. Travers goes at some length into an historical research relative to wounds of the stomach, both *ab interno* and *ab externo*—for which we must refer our readers to the original record. The result of these researches, and an attentive consideration of the examples adduced (if credit can be implicitly attached to them all) would lead one to regard wounds of this viscus as much less dangerous than they are generally supposed to be.—But, for our own parts, we are somewhat more sceptical as to certain wonderful cases on record than Mr. Travers. Does he really believe the story of the two negroes?—If so, Mr. Travers will certainly be saved—for he has abundance of faith. "A lusty young negro man, returning home about noon, went into his house, when seeing some ripe plantains, he eat of them heartily. His father-in-law, about sixty years of age, coming home soon after, and finding the young fellow had eat up his fruit, pulled out his knife, and gave him a desperate wound in the upper region of the belly; *a vast gash being made in the stomach, insomuch, that the plantains which he had eaten burst out through the wound.* The old man immediately fled for it, and the young fellow's companions hearing what was done pursued him. Perceiving them get ground of him, and suspecting their design was to kill him, he pulled out the same knife with which he had stabbed the other, and gave himself a desperate wound also in the upper region of the belly, his stomach being likewise seen, only with this difference, that the last wound was transverse, or from left to right, the first directly up and down; the old fellow was carried home, and lay in the same house where the other lay. This happened about noon, and Mr. Forest the surgeon, came not to dress them till between four and five; *he stitched up both their stomachs entirely*, and their bellies too, except only a small hole for suppuration; a fever seized each of them, and held them about a fortnight. The wounds were brought to a good digestion, and in about a month's time the young fellow went abroad, but the old man, who was in most danger, lay something longer; however, they were both perfectly cured, and have been very well ever since, though it is above fifteen years ago." 92.

Now it appears from the above record that Mr. Forest made up for his tardy attendance, when he did arrive. In fact, he was, like the Devil, double diligent—for he sewed up the old man's stomach *entirely* although there does not appear to have been any wound in that organ! No wonder that the poor old fellow was longer in recovering than the graceless son-in-law, whose stomach he gutted of the plantains. In sober earnest, we think, Mr. Travers would have done better by leaving out this wonderful story of the two negroes.

The case of Mr. Dix shrinks into insignificance, compared with some that Mr. Travers has collected. A young lad was gored by a bull to the extent of about three inches on the left side of the abdomen, half way between the spine of the ilium and the border of the ribs. About three feet of intestines protruded, with a portion of mesentery and omentum. Mr. Dix returned the protruded parts with as little delay as possible, cutting off a portion of lacerated omentum. One stitch was applied, and the dressing was completed by adhesive plaster, compresses and bandage. Proper position, quietude, abstinence, copious blood-letting, leeches, anodynes, injections, and gentle aperients conducted the case to a successful issue, and we have no doubt that Mr. Dix obtained what he certainly deserved, great credit among the inhabitants of Long Buckly, Northamptonshire.—Many a worse managed case has made a man's fortune.

Before taking leave of the subject of gastrotomy, we may mention that, through the kindness of Dr. Blundell, that able and zealous cultivator of medical science, we have had an opportunity of minutely examining the female on whom Mr. Lizars operated successfully for the removal of a diseased ovarium. Our readers will find the case circumstantially detailed between page 337 and page 341 of Number 6 of this Series, for October, 1825. The patient is now in very fair health, all the functions going on regularly (even menstruation) and her flesh progressively increasing in firmness and plumpness. We particularly examined the abdomen, and observed the tremendous cicatrix extending from the scrobiculus cordis to the pubes. The abdomen is still fuller than natural, especially on the left side, where there are some remains of a tumour yet perceptible, both to sight and touch. But it appears that the size of the abdomen generally, and of this side particularly, has remained stationary for some months past, so that there is every prospect of the poor woman's health being sufficiently established to enable her to resume her usual avocations. She is in good spirits, and appears to have suffered nothing from her journey to London. We congratulate Mr. Lizars on this unquestionable proof of the success of his operation—a species of proof which is far from being appended to the generality of similar operations hitherto recorded.

---

27. *Urethro-vaginal Fistula.* In our last number we published a case of this deplorable accident, successfully treated by M. Lallemand of Montpellier, through the means of a suture, resembling that used for hare-lip. We find, by the December Number of our respected cotemporary, the Medical and Physical, that Mr. Hobart, of Cork, has lately performed a somewhat similar operation, and with equal success. The case was a fistulous opening between the meatus urinarius and vagina, in a female, aged 26 years, who had remained ten months incapable of holding her water for a moment, and suffering all the afflicting consequences of excoriation and ulceration of the nates and thighs. On dilating the vagina by means of Mr. Weiss's speculum vaginæ, the aperture, which was of an oval form, (the long diameter in the direction of the axis of the vagina) was discovered, situated about two inches and a half from the os externum, and sufficiently large to admit the point of the finger. Its edges were quite smooth and callous. Mr. Hobart, by means of an ingenious instrument, (a plate of which is given in the journal alluded to) contrived to apply the bloody suture, having first destroyed the edges of the aperture by caustic several times applied. A catheter was, of course, kept in the bladder. Two ligatures were inserted, made of strong silk saturated in wax, to prevent the incrustation of calcareous matter. The ligatures were allowed to remain fourteen days, and were then removed with great facility. In three days afterwards the catheter was withdrawn, when the urine was found to flow along the urethra without any infiltration into the vagina—the sphincter of the bladder recovered its tone in a few days. We hope that, in future, these unfortunate accidents will not be allowed to remain a blot on surgery.

---

28. *Strangulated Hernia with Inflammation.** The valuable time which has been suffered to elapse, and the fruitless efforts which have been made for the reduction of an incarcerated hernia, often place the operating surgeon in a situation of great difficulty, and the patient in extreme danger. We have, on several occasions, drawn the attention of practitioners to the propriety of an early operation in hernia, and the hazards which they run by delay—perhaps we might say by *timidity*. We shall neglect no opportunity of enforcing the measure which we have recommended, because we have seen the danger of inattention to it.

*Case.* Madame S. aged 50, had been of a constipated habit from youth. Sometimes she would go three weeks without a motion, and then would only be relieved by repeated lavements and purgatives. It was doubtless during the strain on one of these occasions, that a portion of gut was forced down under the crural arch, and a hernia produced. The consequences were, acute colicky pains, tension of the abdomen, inclination to vomit, and other symptoms that we need not enumerate. The patient was at a distance from Toulouse, and the complaint was supposed by the practitioner in attendance to be enteritis. Leeches, fomentations, &c. were in vain employed, and stercoraceous vomiting at last induced a suspicion of hernia. An examination soon shewed a descent under the crural arch. The alarm was now great, and no surgeon at Aix or in the neighbourhood would dare to perform an operation for the relief of the poor lady.† It was therefore necessary to send

---

* M. Ducasse, fils. Revue Med. Septembre, 1825.

† This does not speak much for the diffusion of operative surgery through France, where anatomy is so cheaply learned. The words of Ducasse are all the way to Toulouse, and 124 hours elapsed before an operating surgeon could be procured. All this time the hernia was strangulated. The poor lady was now so altered by her sufferings, that she looked absolutely like a corpse. "Sa figure était absolument celle d'un cadavre." The pulse was hardly perceptible—skin cold and clammy —abdomen enormously distended—urine suppressed—vomitings of black and bilious matter—the hernial tumour inflamed and painful to the touch. The little hope that remained depended, of course, on the operation, and this was immediately performed. A portion of omentum had covered the protruded gut, and was completely mortified. It was cut away. The intestine was then exposed, and was quite black, but of pretty firm consistence; it was, therefore, deemed advisable to return it into the abdomen, and close the wound. The vomiting still continued. Castor oil was nevertheless given, and in eight hours after the operation, motions by stool were procured. They were copious, and shewed the castor oil which had been swallowed. The vomitings now ceased, the other symptoms became improved, and the patient fell into a sleep. Next day, however, the vomiting returned—the belly became more swelled—the urine stopped. These symptoms were mitigated by fomentations and proper means, and from this period the patient went on rapidly to convalescence.

This case is worthy of a short record—not as holding out any inducement to delay an operation (for every risk was here run, and no advantage gained by such delay) but to shew that there is some chance of success by an operation, even in cases apparently hopeless. Many surgeons would have refused to operate, under circumstances like the above, when a hernia had been strangulated, or closely incarcerated for more than five days, and when life was fast ebbing. The result, however, shewed the propriety of the operation. But what shall we say to the surgeons of Aix, (a city containing 21,000 inhabitants) who could not muster a man capable of performing an operation for strangulated hernia! Had the fact not been recorded by a French surgeon, we could not have believed it, though we have long been of opinion that the facilities of dissection in Paris did not tend, as might be expected, to make minute anatomists.

---

29. *Supposed Union of fractured Cervix Femoris.** Our readers are aware of the controversy which lately existed relative to the *probability* (for the *possibility* has never we believe been denied) of bony union in

---

remarkable. "Les médécins consultés ne voulurent pas cependant entreprendre une si grave operation. Aucun d'eux n'osa prendre sur lui la responsibilité du traitement d'une maladie dont le bruit s'etait deja repandu *dans toute la contrée*, et tous réclamerènt une main moins timide." Now, notwithstanding the difficulties which are thrown in the way of anatomy in this country, we venture to aver that such a scarcity of surgeons capable of operating, could hardly take place in the smallest village of England, Ireland or Scotland, much less in a place the size of Aix!

* Dr. J. Begbie, Ed. Journ. Med. Science, No. 1.

fracture of the cervix femoris within the capsular ligament. Dr. Begbie has lately published a case in our *junior* northern cotemporary, which bears on this point. A feeble lady, aged 77, slipped on her stairs, and fell on the right hip. On attempting to walk she found she had lost all command over the lower extremity of that side. She was conveyed to bed, but did not suffer much pain. Dr. B. saw her next morning. The limb was shattered about an inch and a half—the knee and toes turned outwards—the heel resting between the malleolus internus and tendo achillis of the other leg. She could not move the limb—all attempts to rotate the thigh occasioned pain in the region of the trochanter major. The limb could not, after being extended, be kept so. No crepitus could be perceived in the recumbent posture. Dr. Abercrombie visited in consultation frequently. The friends were apprised that, from the patient's case, there was little probability of union of the fracture. The treatment was simple—a bandage and pillows. Under this plan she continued about five months, occasionally changing from her bed to her sofa. She then began to make some partial use of her leg, with the assistance of crutches. Afterwards she laid aside the crutches, and walked with the aid of a staff. This she ultimately laid aside, and, by wearing a very high heeled shoe, she was enabled, before the end of the year, to walk with great facility, through the house, and even to descend and ascend the stairs. The knee and foot continued very much erected, and the limb shortened. She was seized with an affection of the brain, and died in April 1824.

On dissection, the capsular ligament was found much thickened—the ligamentum teres healthy. There was a remarkable absorption of the neck of the bone, on the posterior aspect; so that the head was nearly brought into contact with the trochanter major. The bone is preserved in the Museum of the College of Surgeons, Ed. and a drawing given in the Journal. From the drawing, one would be led to suppose that there had been a fracture, and that it had united. The bone itself was sent to Sir Astley Cooper, and on first looking at it, he thought there had been a fracture; but on farther examination he entertained great doubts on this point—first, because " in the internal appearance of the anterior section, there is nothing which has the character of fracture. 2dly. Because there is the most marked appearance of disease around the ligamentum teres:—3dly. Because there is a morbid appearance in the cancelli of each section. Perhaps some diseased change has been produced by the accident." *Letter from Sir Astley.*

Without at all denying the possilbiity of union within the capsule, it is natural to attach considerable importance to the objections which Sir Astley has thus stated to the supposed proofs in the bone subjected to his examination. But we are ready to grant that the knowledge of the previous history gives this case a far greater degree of authenticity than any which we have before seen.. After all, we must be guided in our prognosis by the *probability* and not by the *possibility* of the event. It is needless to observe how rare is the bony union in cases of this kind.

30. *Removal of Cicatrices.** As beauty is only "skin-deep," so the surgeon is often consulted by the fair sex (*male* and female) for the removal of any unsightly spot on so important an organ as the skin, especially that part of it which is exposed to view. In many cases, however, a deformity is also a serious evil, as in those cicatrices about the face and neck, left after burns; and which are by no means to be put down to the score of negligence on the part of the surgeon who first had the charge of the accident. The sore of a burn heals, and all appears to be well; but afterwards it contracts, and (in too many cases at least) irremediable deformity and inconvenience are the results. Sir Astley Cooper appears to think these contractions after burns generally incurable, although Mr. Henry Earle, some years ago, drew the attention of the surgical world to the practice of excision of the cicatrix. Mr. James thinks that, in those cases where the cicatrix exists between the lower jaw and sternum (an occurrence not unfrequent) relief is not commonly obtainable by Mr. Earle's plan. Nevertheless, he himself has been so fortunate as to procure considerable mitigation of the deformity and inconvenience by an operation and subsequent management.

*Case* 1. A girl about nine years of age, had a cicatrix of the kind in question, the consequence of a burn which happened seven months previously. It was large, broad, and tense, tying down the chin closely to the sternum, so that there was not more than an inch of interval. The left corner of the mouth was very much drawn down by it. It also projected much from the ordinary surface of the neck, so as to efface the projection of the chin. On the 9th March, 1824, he performed an operation in the following way.

"I made two incisions, one at either edge of the cicatrix, extending to its termination at the upper part of the sternum where they met; I then dissected up the flap, of which I removed no part, but having completely freed it, I found I was able to dispose of it out of sight under the chin. The distance between the sternum and the chin was now increased to at least three inches by measure.†

"I supported the cicatrix in its situation, first by broad straps of adhesive plaster, and secondly by a compress of lint secured by a broad fillet fastened at the top of the head, so as to make a good chin. I then tried to approximate the edges of the wound beneath by straps of plaster, but this could only be accomplished in a partial manner. I then put on the screw collar, the construction of which is described at the end of this paper. I soon found that this was of no service, for when the wound inflamed and became irritable, she contrived, from its being rather too large, to slip the chin within it. I therefore contented myself with applying a collar of pasteboard with a poultice underneath, and laid her head nearly on the same horizontal line as her body, till a

---

* Mr. James, Surgeon to Devon Hospital, Med. Chir. Trans. Vol. xiii.

"† It is generally necessary to set free the edges of the wound by little transverse cuts, which was done in this and the succeeding case."

smaller screw collar could be made. In the mean time suppuration was freely established, and the irritation of the wound having subsided, I was enabled after a few days to apply this.

"In the course of the cure this girl was attacked by measles, which somewhat delayed it; notwithstanding which, the sore healed in about four months. A similar period has since elapsed, during which, instead of any ground having been lost, I think the parts have given way still more, and the distance between the chin and sternum is now three inches and a half. The old cicatrix is perfectly concealed under the chin, and as she grows up I think very little trace will remain of this deformity." 155.

*Case* 2. This was a girl of about 13 years of age, who had been burnt four years previously. The cicatrix was more extensive than in the first case, reaching as far as the lower edge of the second rib, being exceedingly broad and dense on the thorax, but less so on the front of the neck. The chin was drawn very near the sternum; and was effaced. The operation in this case varied somewhat from the one just described.

"It would have been a dreadfully severe, and probably a much less serviceable operation, to have dissected off the whole of this immense cicatrix, and therefore I adopted the following plan: I made two incisions, one on either edge of the cicatrix, of about three or four inches; I then pinched up the cicatrix in the middle between the finger and thumb of my left hand and drew it forcibly out; I next pushed a long, straight-backed, narrow knife through from one incision to the other, and turning its blade outwards, I at once divided the intervening band, making a cut of at least two inches with very trifling pain; I now freely detached both flaps, until the girl could carry her chin horizontally without dragging the integuments of the thorax." 156.

"I proceeded as before to tuck the upper flap under the chin, and confined it in its situation by long straps, compress and broad fillet. Then, having dressed the neck below lightly, retaining the lower flap in the situation to which it had been separated from the other (a distance of more than two inches), I applied a poultice, and over that a pasteboard collar; and I continued this plan for some days, until the chin was formed and suppuration established. I then substituted broad straps in front of the throat, for the pasteboard collar, and applied the screw collar with a short screw. By degrees we were enabled to raise the chin to the full extent of this screw, and have since substituted a longer one.

"The result of this operation has been equally successful as the last; the chin is well formed, and the new cicatrix is soft and pliant. The girl can raise her chin without difficulty above the horizontal line, and the measured distance, without stretching the integuments, is three inches and a half. The neck has now been well sixteen weeks. In this case I continued a poultice in a bag over the other dressings nearly to the termination of the cure, and with advantage." 157.

The question now is, will the new cicatrices contract? Mr. James thinks not—but we confess that we have our doubts. Time will tell —and Mr. James will, we are assured, give the necessary information to the public. As a useful precaution, Mr. J. intends that these girls shall wear their collars for a year or two longer. It is obvious that this operation differs somewhat from that proposed and practised by Mr. Earle. The latter is of opinion, that the advantage is derived from approximating the edges of the wound, and so obtaining a cicatrix in the transverse, instead of the longitudinal direction. The same object, Mr. James thinks, will be obtained by the natural process above-mentioned, if care be taken to maintain a proper distance in a longitudinal direction. In fact, the after-treatment is a *sine quâ non* in the obtainment of success.

Mr. James deserves the thanks of his brethren for making known these cases. We hope they will prove useful to the surgical portion of the profession.

---

31. *Injury of the Head.* Mr. Oswald (Isle of Man) was called to a man, who, on the preceding day, had fallen from his horse, and was completely stunned. He had been bled and carried home, a distance of eight miles, still in a state of insensibility. Mr. Oswald found a puffy tumour on the occiput, which was laid open, but no fracture discovered. The kind of insensibility is thus described by Mr. O. "At short intervals, the patient appeared to sleep composedly, with half-closed eyes; and as often was very restless, changing his position, tossing his arms about the bed and over his head, and sometimes snoring with a heavy inspiration or sigh. But his breathing was not stertorous: on the contrary, it was for the most part placid, or more than usually low and easy. The eyelids were at all times partly closed, and there was a dulness and fixedness in the eye itself. The pupil contracted readily when a lighted candle was held before it; and he put up his hand to push the candle away, as if it gave him pain. He had swallowed nothing since the accident (now nineteen hours), and was not only incapable of doing so,but sometimes showed an aversion to the attempt. The power of speech was also lost, and he paid no attention to questions put to him in a loud voice. His pulse beat from 76 to 84 in the minute, being sometimes irregular, and somewhat sharp and wiry; but when he was roused by any effort, such as an attempt to make him swallow, or by an examination of the head, it immediately rose to 96 and 100, and upwards, according to the degree of excitement; and withal beat more feebly, and was less wiry than when at its lowest rate. His skin was cool. He passed water freely during the night, and, when inclined to do so, felt for the pot, as a person would do in the dark. In short, he appeared much like one just upon the verge of total insensibility from intoxication, only there was more paleness than flushing of the face, and an expression of greater distress in his whole manner and appearance." 371. Mr. O. enlarged the previous incision in a crucial manner, but still could discover nothing of fracture. Three dozen of leeches to the occipital region. Purgative enemata in succession till the bowels were relieved. The pulse was improved, and the restlessness diminished; but the insensibility continued. In the evening of the second day, the mouth and tongue being parched, and covered with sordes, a blister was applied to the nape of the neck. *Third day.* Still insensible—pulse 80—can swallow nothing—injection of pabulous fluids per anum—36 leeches to the head. Some milk and tea thrown into the stomach by means of a machine. In this operation, he manifested symptoms not indicative of the great degree of insensibility above described, for *he grasped the tube, on its first introduction, and forcibly withdrew it from the stomach,* making a loud but inarticulate noise at the same time. After this effort his face became suffused, and his pulse rose to 125; but he soon fell back into his former state of quiescent stupor and insensibility. *Fourth day.* Power of speech and deglutition in some degree restored. Often repeats in a childish way, that he is very ill—complains of oppressive head-ache. 36 leeches to the head—purgative of jalap and calomel. In the evening of this day there were symptoms of reaction, and upwards of 40 ounces of blood were abstracted from a vein, in full stream, when the head-ache became easier, and an approach to syncope took place. *Evening.* The symptoms improved. In two or three days he was convalescent, and soon completely recovered.

We agree with our author, that this was a case of concussion of the brain, but can hardly go so far as to think with him, that the patient would "probably never have recovered, had not injection into the stomach been practised, so as to rouse re-action in that organ and consequently in the whole system." On the contrary, we think the injection into the stomach of a little milk and tea could have done no good—and the force which the patient exerted at the time, proved that muscular power was not at a very low ebb. We are disposed to think that depletion, in this case, was employed full early. In concussion, we should wait till some degree of reaction is manifested, and then our depletive measures will tell to more advantage. They are embarrassing accidents, however, and we are far from wishing to cavil with the treatment pursued by Mr. Oswald. It was successful—which is not a little in its favour.

